# Coat-colour-related genotypes, phenotyping and biometric assessment of three ecotypes of pigs in Cameroon

**DOI:** 10.5194/aab-68-239-2025

**Published:** 2025-03-28

**Authors:** Aveniol Idelo Nono Ekane, Ferdinand Ngoula, Blaise Arnaud Hako Touko

**Affiliations:** 1 Biotechnology and Bioinformatics Research Unit, Department of Animal Science, Faculty of Agronomy and Agricultural Sciences, University of Dschang, Dschang, Cameroon; 2 Animal Physiology and Health Research Unit, Department of Animal Science, Faculty of Agronomy and Agricultural Sciences, University of Dschang, Dschang, Cameroon

## Abstract

A study was conducted from June 2022 to December 2023 in three agroecological zones in Cameroon to describe the local pigs and to develop a standard method for reporting their phenotypic-diversity information. A systematic sample of 152 adult pigs was considered and described in terms of coat colour major genes and body measurements. The results show that local pigs are heterogeneous in colour and are predominantly white (34.2 %). The melanocortin 1 receptor gene (MC1R) was the most significant gene for primary coat colour, with five alleles, including E^+^ (wild type), E (dominant black), E^*d*^ (black with white belt), E^p^ (white with black spots) and e (recessive red). The E^p^ allele was the most predominant at 75 % allele frequency. The agouti signalling protein (ASIP) gene was the only secondary coat colour determiner observed, with its recessive non-agouti allele (a) present (100 %). The coat colour modifier is the tyrosine-kinase protein gene (KIT), with six alleles, including I (colour inhibition), I^*d*^ (green), I^*p*^ (white and black spots), I^*b*^ (white belt), i^*m*^ (dirty white) and i (non-kit), with the predominant allele being I^*p*^ (39.5 %). The agroecological zone influenced body measurements, including head length, between-ear distance, snout size and ischium width ( P < 0.05). Furthermore, the highest live weight of 82.47 ± 23.86 kg was obtained in the monomodal-rainfall forest (MRF) zone. The biometrical structure shows that the local Cameroon pigs can be organised into three subgroups. This study provides the broader audience with valuable insights into the phenotypic diversity of the local Cameroon pigs and a standardised description and reporting protocol.

## 1 Introduction

The success of agriculture and livestock farming in most countries largely depends on utilising local resources to increase production. These resources have unique characteristics that are not commonly found elsewhere. Regarding livestock farming, sociological factors such as consumer preference for the colour of an animal's coat or plumage play a significant role in animal protein consumption. For instance, in Korea and many other Asian countries, consumers prefer coloured pigs over uncoloured ones (Choy et al., 2002). This makes the colour of an animal's coat critical in identifying specific breeds or crosses, similarly to blood and biochemical or molecular markers. As a result, there is a renewed interest in coat colour variations as they can help identify certain breeds or specific crosses (Legault and Chardon, 2000).

In Cameroon, the pig industry is the third largest sector of animal production, contributing over 53 877 t of meat (INS, 2019). Pigs are highly adaptable to various management and feeding conditions, and they proliferate, reach sexual maturity early, and have high prolificacy and efficient feed conversion. These qualities make them a popular choice as a livestock species (Taverner and Dunkin, 1996). Locally, the Cameroonian pig has gained recognition for its low fat percentage and disease resistance. However, the need for knowledge about this genetic resource makes it difficult for researchers, breeders and consumers to choose the best option. A phenotypic description based on the significant colouration genes could significantly benefit these users. Certain aptitudes and diseases have been linked to the colouration (Jonathan, 2003; Hutu et al., 2020; Hutt, 2022).

It is widely believed that most domestic pig breeds are black because they carry a recessive allele called mono agouti (a) at the agouti locus and a normal or wild allele (E) at the extension locus. This theory suggests that the Large White boar and wild boar have the following genotypes at the dominant white, agouti and extension loci: I/I, a/a and E^*P*^/E^*P*^ and i/i, A/A and E^+^/E^+^, respectively (Ollivier and Sellier, 1982). Describing animal resources based on phenotype and genotype is efficient and cost-effective. In Cameroon, the socio-economic and phenotypic characterisation of local pig breeds has been carried out in various regions, including the Sudano–Sahelian region, humid forests with bimodal rainfall, the Western Highlands, the Sudano Guinea zone and humid forests with monomodal rainfall (Motsa'a et al., 2017; Ghomsi et al., 2022; Siewé et al., 2021). However, some unique pig ecotypes, such as the Dimako pig in humid forests with bimodal rainfall, have not been considered despite the morphological and biometrical description achieved so far. Moreover, without a standard description and reporting protocol, transferring information on genetic variability is challenging for multi-allelic genes because of the high number of gene combinations resulting in phenotype variability. This is also true for pig populations, where three genes (MC1R, ASIP and KIT) have four to seven alleles each and interact with three reported gene dilutions of two to three of these (Fontanesi and Russo, 2013).

The present study was carried out to remedy this shortcoming. Its main aim is to contribute to a better understanding and reporting of animal genetic resources and to provide a standard description and reporting protocol to a broad scientific audience.

## 2 Materials and methods

### 2.1 Field study location

**Figure 1:**
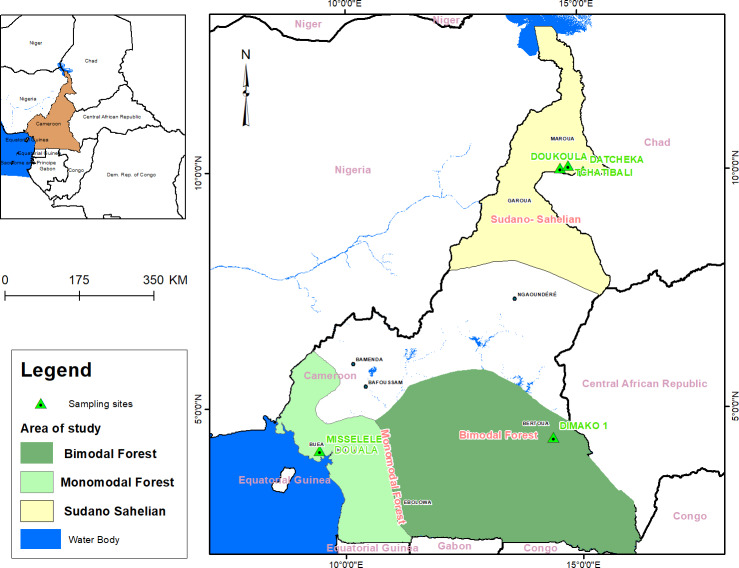
Map of the agroecological zones and sample collection sites in Cameroon.

The study was conducted in Cameroon from June 2022 to December 2023 in three agroecological zones: the bimodal-rainfall forest zone (BRZ), the monomodal-rainfall forest zone (MRZ) and the Sudano–Sahelian zone (SSZ) (Fig. 1). Based on WorldClim data (2024), the SSZ is the hottest zone, with 7 to 9 months of dry season and a maximum of 5 months of rainy season. Relative humidity varies from low to high (20 % to 80 %). It is situated between 8°3^′′^ and 12°5^′′^ N and 12°3^′′^ and 15°4^′′^ E. The rainfall intensity varies from 350 to 800 mm annually, with August being the rainiest month (195 mm of rainfall). April is the hottest month (26 to 40 °C ). The BRZ is located between 2°^′′^ and 4°5^′′^ N and 10°3^′′^ and 16°1^′′^ E. It is characterised by a long rainy season of 10 months, from early February to mid-December, with two peaks in May (118 mm rainfall) and September (197 mm rainfall) and with 1560 mm yearly. The dry season is short (merely 2 months). The annual temperature ranges from 17 to 32 °C , with February being the hottest month (19–32 °C ). The relative humidity varies from 67 % to 87 %. The MRZ is located between 44°10^′^00^′′^–5°50^′^00^′′^ N and 9°30^′^00^′′^ E. Annual rainfall is high (2500 to 4000 mm ), with July being the rainiest month (700 mm in Buea). The dry season is 3.3 months, from January to April, with February being the hottest month (26 to 32 °C ). Relative humidity is high throughout the year (69 % to 87 %).

### 2.2 Animal material

The local pig populations of Cameroon are under-exploited and not numerically abundant, representing less than 10 % of the national pig population, which is dominated by commercial foreign breeds. They are small-sized animals mostly reared extensively in low-input systems due to their small-flock-size families (3 to 20 pigs), with sows having a litter size ranging from one to five. There is limited to no information on characteristic such as weight or housing, making it difficult to carry out characterisation studies (Motsa'a et al., 2021). The sample used was heterogeneous in terms of coat colours and was made up of adult pigs of both sexes, aged 8 months and more. Gestating sows were not sampled, and for genetically related pigs (pigs sharing an identical sire and dam), only one pig per coat colour type was considered.

### 2.3 Sampling and data collection

A sampling was conducted under the facilitation of the extension services of the Ministry of Livestock, Fisheries and Animal Industries (MINEPIA) to identify all possible variants of local pigs based on coat colour in each of the three agroecological zones. The sampling involved selecting 152 adult pigs representing 10 % of the pigs owned by 52 households rearing local pigs as their secondary or tertiary activity.

#### 2.3.1 Phenotyping and identification code system

Phenotyping consisted of describing a pig based on identified significant gene loci, allelic series and visible trait characteristics. Different combinations of the major genes of a phenotype were named “morphotypes”. Each major gene was identified from its expression, and the chromosome number and corresponding locus information were obtained from the NCBI databank or credible literature sources. The major gene variants considered for the description of the morphotypes were those relating to coat colour (enhancers, dilutions and modifiers) according to Ollivier and Sellier (1992). The phenotyping was done according to Fontanesi and Russo (2013).

The characteristics of the major genes, the locus involved, their alleles, and the expressed coat colour and modifications are presented in Table 1. The coding of the phenotypic and genetic information was done according to a CL-5N system, where

- C is the country initial (CMR for Cameroon);
- L is the locality initial, with SSZ representing the Sudano–Sahelian zone, BRZ representing the bimodal-rainfall forest, and MRZ representing the monomodal-rainfall forest;
- the first N is the most expressed gene allele of the primary coat colour in the phenotype (E^+^, E, E^p^, E^*d*^, e);
- the second N is to be maintained if the secondary coat colour is absent or replaced by the corresponding gene initials (A^*w*^, A^*b*^, a, a^*s*^);
- the third N is to be maintained if the coat colour modifier is absent or replaced by gene alleles of the modifier (I, I^*d*^, I^*p*^, I^*b*^, I, i^*m*^);
- the fourth N is to be maintained if the coat colour enhancer or dilution is absent or replaced by gene alleles of the enhancer or dilution (D, d^*s*^, d^*p*^);
- the fifth N is to be maintained if the coat colours of white belts or white heads are absent or replaced by the corresponding alleles (Be^*w*^; be; be^*b*^ or He; he).

**Table 1:** Characteristics of the major genes used for the phenotyping of indigenous pigs.

Coat colourtype	Gene	Symbol	Locus	Chromosome	Alleles	Description	Source
Primarycoat colour	Melanocortin 1receptor	MC1R	Extension	6	E^+^, E, E^*d*^, E^*P*^, e	Wild type, dominant black, white belt, black spotted, recessive red	Fontanesi andRusso (2013)
Secondarycoat colour	Agouti signallingprotein	ASIP	Agouti	17	A^*w*^, A^*b*^, a, a^*s*^	White-bellied agouti, badger-head agouti, non-agouti, sepia	Fontanesi andRusso (2013)
Coat colourmodifiers	Tyrosine-kinase protein gene	KIT	DominantWhite	8	I, I^*d*^, I^*p*^, I^*b*^, i, i^*m*^	Inhibition colour, green, black spotted or white, white belt, normal colour, dirty white	Fontanesi andRusso (2013)
Coat colourdilution	Dilution gene	D	–	–	D, d^*s*^, d^*p*^	Normal, sepia, recessive dilution	Legault andChardon (2000)
Other colours	White-belt gene	Be	White belt	–	Be^*w*^, be, be^*b*^	White belt, lack of white belt, half colour and white head	Legault and Chardon (2000)
	White head	He	White head	–	He, he	White head, normal	

#### 2.3.2 Biometric assessment and genetic frequency estimation

**Figure 2:**
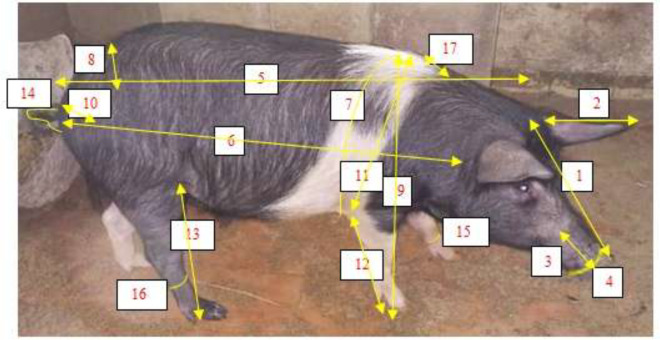
Biometric measurements of the local pig breeds. Head length (1), ear length (2), muzzle length (3), muzzle circumference (4), body length (5), scapuloischial length (6), chest circumference (7), wither height (8), hip width (9), ischium width (10), wither height (11), front leg length (12), hind leg length (13), tail length (14), cannon circumference (15), ham circumference (16), hair length (17).

The biometric measurements were obtained using a graduated metre according to the protocol described by FAO (2013). A total of 19 body measurements were then assessed, as illustrated in Fig. 2.

The live body weights were determined from the chest circumference, which is best predictor according to Santolini (2004), using the following barometric equation:

1 $$Y=0.0129x^{2}-0.6209x+10.778;R^{2}=0.965,$$

with Y being the body weight, X being the chest circumference, and $R^{2}$ being the determination coefficient or precision.

The frequencies of the various phenotypes, genotypes and alleles were estimated as follows:

2Phenotype frequency (Fi)=number of individuals with  phenotypes ”i”total number of phenotypes  in the sample,3Genotype frequency (Fj)=number of individuals with  genotype ”j”total number of genotypes  in the sample,4Allele frequency (Fk)=number of allele ”k”  in the sample2×(total number of individuals  in the sample).

### 2.4 Data analysis

Descriptive statistics were used to calculate the various measurements' means, standard deviations and coefficients of variation. An analysis of variance was conducted to investigate the impact of the ecotype, coat colour and genetic type on measurements. Pearson's correlation coefficients were utilised to evaluate the degree and significance of correlation between the measurements. Principal component analysis (PCA) was performed based on the measurements to identify the main criteria explaining the morphometrical variability. A discriminant factor analysis (DFA) based on 17 body measurements, coupled with the hierarchical ascending classification (HAC), was conducted to determine the clusters of individuals in the population according to their resemblance to their biometrical measurements. The statistical analyses were conducted using SPSS 21.0 and XLSTAT 2022 software.

## 3 Results

### 3.1 Phenotyping of local pigs in the three agroecological zones of Cameroon

#### 3.1.1 Frequency distribution of coat colours

**Figure 3:**
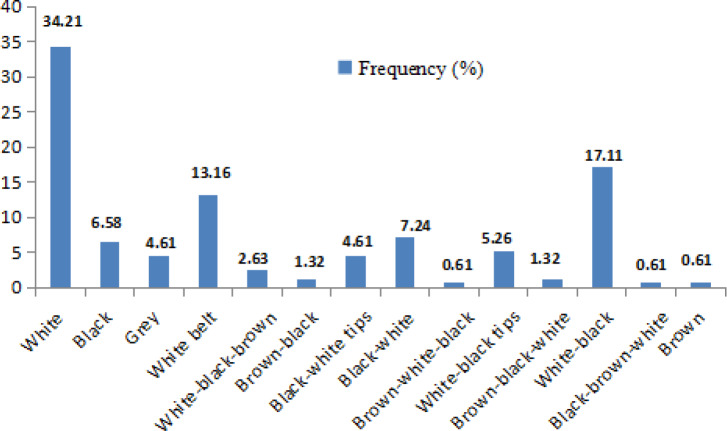
Frequency distribution of coat colours recorded in the local pig breeds in Cameroon.

The frequency distributions of the coat colours of local pig morphotypes are presented in Fig. 3. The most common uniform colour pattern is white (34.21 %), followed by black (6.58 %), grey (4.61 %) and brown (0.65 %). The most usual colour pattern combinations are white–black (17.11 %), followed by black with a white belt (13.16 %) and eight other combinations (23.79 %).

#### 3.1.2 Morphotype description and coding

The phenotypes and the corresponding genotypes and CL-5N codes of pigs for the coat colour genes are presented in Table 2. A total of 14 morphotypes are described, with 3 genes being responsible for primary (MC1R) and secondary coat colours (ASIP and KIT). The MC1R gene identified has four allele series present (E, E^p^, E^*d*^ and e) out of the four known so far. The ASIP gene has two (A, a) out of the four known so far, and the KIT has four (I, I^*d*^, I^*b*^ and i) out of the six known so far. Two gene modifiers are responsible for the white coat colour pattern (white belt and white head). The extension locus of the MC1R gene is the primary coat colour gene observed in all the pigs, either being homozygote dominant (EE) and associated with spotting (E^p^E^p^), having a white belt (E^*d*^E^*d*^), or being heterozygotic due to the recessive allele form (e).

**Table 2:** Morphotype codes and descriptions of their phenotypes and genotypes.

Morphotypes and code	Coat colour (phenotype)	Genotypes at the five loci					Frequency
		Agouti	Dominant	Extension	White head	White belt	
		(A)	white (I)	(E)	(He)	(Be)	
CMREN-ENNNN (A)	Black	aa	II	EE	hehe	bebe	6.50
CMRES-E^*P*^NINN (B)	White	aa	II	E^*P*^E^*P*^	hehe	bebe	34.21
CMRFN-ENI^*d*^NN (C)	Grey	aa	I^*d*^I^*d*^	EE	–	–	4.60
CMREs-E^*p*^NNNN (D)	Brown–black	aa	I^*p*^i	E^p^E^p^	hehe	bebe	1.31
CMRFN-E^*p*^NI^*b*^NBe (E)	White belt	aa	I^*b*^I^*b*^	E^*d*^E^*d*^	hehe	Be^*w*^be	13.15
CMRFN-E^*p*^NI^*p*^NN (F)	Black–white	aa	I^*p*^I^*p*^	E^p^E^p^	hehe	bebe	7.20
CMRFN-E^*P*^NI^*p*^NN (G)	White–black	aa	I^*p*^I^*p*^	E^p^E^p^	hehe	bebe	17.10
CMREs-E^*P*^NI^*p*^NHe (H)	White–black tips	aa	I^*p*^I^*p*^	E^p^E^p^	HeHe	bebe	5.26
CMRSO-E^*p*^NI^*p*^NHe (I)	Black–white tips	aa	I^*p*^I^*p*^	E^p^E^p^	HeHe	bebe	4.60
CMRFN-E^*P*^NI^*p*^NN (J)	Brown–black–white	aa	I^*p*^I^*p*^	E^p^E^p^	hehe	bebe	1.31
CMRSO-E^*P*^NI^*p*^NN (K)	Brown–white–black	aa	I^*p*^I^*p*^	E^p^E^p^	hehe	bebe	0,65
CMRFN-E^*P*^NI^*p*^NHe (L)	Black–brown–white	aa	I^*p*^I^*p*^	E^p^E^p^	HeHe	bebe	0.65
CMRSO-E^*p*^NI^*p*^NHe (M)	White–black–brown	aa	I^*p*^I^*p*^	E^p^E^p^	HeHe	bebe	2.63
CMR-SO-eNNNN (N)	Brown	aa	II	ee	hehe	bebe	0.65

**Figure 4:**
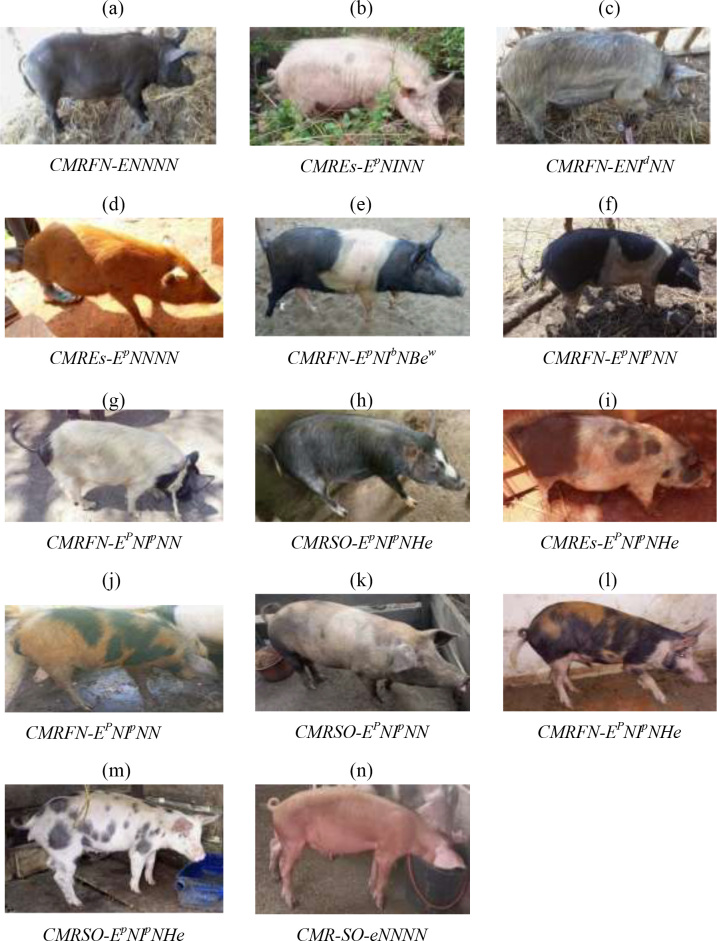
Picture and morphotype code highlighting the genes for primary, modifier and other feathering colours in pigs at the MC1R locus, agouti locus and extension locus. Shown for the MC1R: E (dominant black), E^*d*^ (black with white belt), E^p^ (black-spotted white) and e (recessive red). The modifier is the tyrosine kinase protein gene (I for colour inhibition, I^*d*^ for green, I^*p*^ for white and black spots, and I^*b*^ for the white belt). Also shown are the white belt (Bew) and the white head (He). Also shown: the white belt (Be^*w*^) and white head (He).

Of the 14 morphotypes identified (Table 2), 7 are located in the far north, 3 are located in the east, and 4 are located in the southwest. It can be noticed that different morphotypes may have the same colour combinations but in various proportions, dilutions and distributions, as observed in the morphotypes J, K, L and M, which are all brown, white and black colour combinations. The morphotype pictures and the corresponding standard coding system are presented in Fig. 4.

#### 3.1.3 Phenotype, genotype and allele frequencies of coat colour genes

#### 3.1.4 Phenotype frequency

Table 3 shows the frequency distribution of the phenotypes of the local pigs in the three agroecological zones of Cameroon. The white pig is the most common, with the highest frequency observed in the BRZ (57.5 %) and then in the MRZ (51.16 %) and the SSZ (16.4 %). The black pig comes second among monochromes, with 12.5 % in the BRZ and then 4.96 % in the SSZ and 3.22 % in the MRZ. The grey pig was only present in the SSZ (4.60 %), whereas the brown pig was observed only in the BRZ (2.5 %). The most common colour combinations included the black–white-belt phenotype at 22.22 % and 2.5 % in the SSZ and BRZ, respectively, followed by the white–black phenotype (32.69 %) observed only in the SSZ.

**Table 3:** Phenotype frequency of the local pigs according to the agroecological zone.

Phenotype	Agroecological zone						Total	
	BRZ		MRZ		SSZ			
	n	%	n	%	n	%	n	%
Black	5	12.5	1	3.22	4	4.93	10	6.50
White	23	57.5	16	51.61	13	16.04	52	34.21
Grey	0	0	0	0	7	8.64	7	4.60
Brown–black	0	0	2	6.45	0	0	2	1.31
White belt	2	5	0	0	18	22.22	20	13.15
Black–white	1	2.5	0	0	10	12.34	11	7.20
White–black	0	0	0	0	26	32.09	26	17.10
White–black tips	5	12.5	3	9.67	0	0	8	5.26
Black–white tips	0	0	7	22.5	0	0	7	4.60
Brown–black–white	0	0	0	0	2	2.46	2	1.31
Brown–white–black	0	0	1	3.22	0	0	1	0,65
Black–brown–white	1	2.5	0	0	0	0	1	0.65
White–black–brown	2	5	1	3.22	1	1.23	4	2.63
Brown	1	2.5	0	0	0	0	1	0.65
Total	40	100	31	100	81	100	152	100.00

n denotes sample size, (%) denotes phenotype frequency, BRZ denotes the bimodal-rainfall forest zone, MRZ denotes the monomodal-rainfall forest zone, and SSZ denotes the Sudano–Sahelian zone.

#### 3.1.5 Genotype frequency

Table 4 presents the genotype frequency of the indigenous pigs of Cameroon for the five loci of interest. Only 10 genotypes were obtained for the 14 phenotypes as four different morphotypes presented the same genotype, including black–white (F) and white–black (G); white–black tips (H) and black–white tips (I); brown–black–white (J) and brown–white–black (K); and, finally, black–brown–white (L) and white–black–brown (M). The most frequent genotype (aa/II/E^*P*^E^*P*^/hehe/bebe was monochrome white (34.21 %). The least frequent genotype (aa/ii/Ee/hehe/bebe) was monochrome brown (0.65 %).

**Table 4:** Genotype frequency of the indigenous pig samples from three agroecological zones of Cameroon.

Phenotype	Genotype					Total	
	A	I	E	He	Be	n	%
Black	aa	ii	EE	hehe	bebe	10	6.50
White	aa	II	E^*P*^E^*P*^	hehe	bebe	52	34.21
Grey	aa	I^*d*^I^*d*^	EE	hehe	bebe	7	4.60
Brown–black	aa	ii	E^*P*^E^*P*^	hehe	bebe	2	1.31
White belt	aa	I^*b*^I^*b*^	E^*d*^E^*d*^	hehe	Be^*w*^be	20	13.15
Black–white	aa	I^*p*^I^*p*^	E^p^E^p^	hehe	bebe	37	24.30
White–black tips	aa	I^*p*^I^*p*^	E^p^E^p^	HeHe	bebe	15	5.26
Brown–black–white	aa	I^*p*^I^*p*^	E^*P*^E^p^	HeHe	bebe	3	1.96
Black–brown–white	aa	I^*p*^I^*p*^	E^*P*^E^p^	HeHe	bebe	5	3.28
Brown	aa	Ii	ee	hehe	bebe	1	0.65
Total						152	100

#### 3.1.6 Allele frequency

Table 5 shows the allele frequency of the local pig population. A total of 12 alleles of coat colour and modifiers were observed, including 4 alleles for the primary coat colour at the MC1R locus, 1 allele for secondary colours at the ASP locus, 4 alleles for modifiers at the KIT locus and finally 4 alleles for colour enhancers or dilutors at undescribed loci. The allele with the highest frequency is E^p^ (75.00 %), and the allele with the lowest frequency is e (0.65 %). The melanocortin 1 receptor (MC1R) locus is the most determining coat colour gene, with four alleles present (E, E^p^, e and E^*d*^) at 11.18 %, 75.00 %, 0.65 % and 13.15 %, respectively.

**Table 5:** Allele frequencies in terms of coat colour genes of the local pig population.

Locus	Alleles	Total	
		n	%
Primary colour	E	34	11.18
MC1R	E^p^	228	75.00
	E^*d*^	40	13.15
	e	2	0.65
	Total	304	100.00
Secondary colour	A	00	00.00
ASIP	a	304	100.00
	Total	304	100.00
Modifier	I	104	34.21
KIT	I^*d*^	14	4.60
	I^*p*^	120	39.47
	I^*b*^	40	13.15
	i	26	8.55
	Total	304	100.00
Others	He	40	13.15
	he	264	86.84
	Total	304	100.00
	Be^*w*^	40	13.15
	be	264	86.84
	Total	304	100.00

### 3.2 Biometrical characteristics of the local pigs of Cameroon

#### 3.2.1 Body measurements of local pigs of Cameroon

Table 6 shows that most of the biometric measurements were influenced by the agroecological zone ( P < 0.05), except for the length of the head, the length of the snout and the ischium width ( P > 0.05). Furthermore, the highest live weight (82.47 ± 23.86 kg ) was obtained in the MRZ. The coefficient of variation suggests that the population of local pigs in Cameroon is relatively dispersed for almost all 17 variables.

**Table 6:** Biometric characteristics (mean and coefficient of variation) of local pig populations in Cameroon according to agroecological zone.

Variable	Agroecological zone							
	Bimodal-rainfall forest		Monomodal-rainfall forest		Sudano–Sahelian		Mean and SD	
	( N=40 )		( N=31 )		( N=81 )		( N=152 )	
	$\bar{X}\pm \mathrm{SD}$	CV	$\bar{X}\pm \mathrm{SD}$	CV	$\bar{X}\pm \mathrm{SD}$	CV	$\bar{X}\pm \mathrm{SD}$	CV
HL	25.76 ± 5.28^a^	20.50	27.29 ± 3.88^a^	14.22	27.75 ± 2.40^a^	8.53	27.13 ± 3.75	14.26
EL	14.26 ± 5.71^a^	40.04	18.53 ± 2.09^c^	11.28	15.99 ± 2.92^b^	18.26	16.05 ± 3.99	24.86
ML	10.71 ± 2.72^a^	25.40	11.37 ± 1.74^a^	15,30	10.62 ± 1.35^a^	12.71	10.8 ± 1.89	17.50
MC	12.48 ± 2.82^a, b^	22.60	13.24 ± 1.43^b^	10.80	12.37 ± 1.55^a^	12.53	12.58 ± 1.96	15.58
BL	69.43 ± 18^a^	25.93	80.06 ± 10.62^b^	13.27	68.06 ± 7.52^a^	11.05	70.87 ± 12.58	17.75
SIL	58.44 ± 15.54^a^	26.59	67.84 ± 8.82^b^	13.00	58.37 ± 7.33^a^	12.56	60.32 ± 10.99	18.22
CC	87.13 ± 18.49^a^	21.22	101.48 ± 12.17^b^	11.99	88.36 ± 9,72^a^	11.00	90.71 ± 14.07	15.51
HW	16.21 ± 2,88^a^	17.77	17.44 ± 2.76^b^	15.83	15.69 ± 2.37^a^	15.11	16.18 ± 2.66	16.44
WH	55.34 ± 14.49^a^	26.18	63.29 ± 7.1^b^	11.22	56,43 ± 6.79^a^	12.03	57.54 ± 9.87	17.15
IW	8.93 ± 2.31^a^	25.85	9.42 ± 1.73^a^	18.37	8.64 ± 1.34^a^	15.51	8.99 ± 1.79	19.91
CD	29.39 ± 6.25^a^	21.27	33.03 ± 3.81^b^	11.53	28.46 ± 3.29^a^	11.56	29.63 ± 4.67	15.76
FLL	26.63 ± 8.71^a^	32.71	32.39 ± 3.13^b^	9.66	27.26 ± 3.08^a^	11.30	28.14 ± 5.6	19.90
HnL	31.2 ± 10.92^a^	35.00	34.61 ± 3.59^b^	10.37	30 ± 3.94^a^	13.13	31.26 ± 6.69	21.40
TL	25.68 ± 10.04^a^	39.10	29.55 ± 5.19^b^	17.56	24.36 ± 3.84^a^	15.76	25.77 ± 6.59	25.57
CaC	13.56 ± 3.06^a^	22.57	14.82 ± 1.91^c^	12.89	12.62 ± 1.32^a^	10.46	13.32 ± 2.19	16.44
HaL	5.29 ± 1.84^b^	34.78	3.79 ± 1.43^a^	37.73	5.96 ± 1.71^a^	28.69	5.34 ± 1.88	35.21
BW	58.9 ± 30.66^a^	52.05	82.47 ± 23.86^b^	28.93	57.83 ± 16.18^a^	27.98	63.14 ± 24.31	38.50

^a^, ^b^, ^c^, ^d^ The values which have the same letter in the same column were comparable statistically ( P < 0.05). Head length is denoted by HL, head height is denoted by HH, ear length is denoted by EL, ear width is denoted by EW, muzzle length is denoted by ML, muzzle circumference is denoted by MC, body length is denoted by BL, scapuloischial length is denoted by SIL, chest circumference is denoted by CC, hip width is denoted by HW, wither height is denoted by WH, ischium width is denoted by IW, chest depth is denoted by CD, front leg length is denoted by FLL, hind leg length is denoted by HnL, tail length is denoted by TL, cannon circumference is denoted by CaC, ham circumference is denoted by HC, hair length is denoted by HaL, and body weight is denoted by BW.

#### 3.2.2 Correlation between body measurements

Table 7 shows the correlation between body measurements. Strong and weak correlations were observed here and there, being either positive or negative, with most of them being significant at 5 %. The strongest ( r=0.9035 ) and most significant ( P < 0.05) one was found between body length (BL) and scapula ischial length (SIL).

**Table 7:** Correlation matrix (Pearson ( n )) between measurements of local pigs in Cameroon.

Variable	HL	EL	ML	MC	BL	SIL	CC	HW	WH	IW	CD	FLL	HL	TL	CaC	HaL	BW
HL	1																
EL	0.56^∗^	1															
ML	0.70^∗^	0.56^∗^	1														
MC	0.58^∗^	0.54^∗^	0.58^∗^	1													
BL	0.70^∗^	0.68^∗^	0.68^∗^	0.66^∗^	1												
SIL	0.69^∗^	0.69^∗^	0.67^∗^	0.62^∗^	0.90^∗^	1											
CC	0.67^∗^	0.64^∗^	0.65^∗^	0.64^∗^	0.84^∗^	0.82^∗^	1										
HW	0.47^∗^	0.40^∗^	0.42^∗^	0.44^∗^	0.59^∗^	0.57^∗^	0.67^∗^	1									
WH	0.73^∗^	0.72^∗^	0.69^∗^	0.68^∗^	0.85^∗^	0.83^∗^	0.82^∗^	0.57^∗^	1								
IW	0.19	0.21^∗^	0.30^∗^	0.29^∗^	0.20^∗^	0.25^∗^	0.21^∗^	0.20	0.30^∗^	1							
CD	0.64^∗^	0.59^∗^	0.66^∗^	0.67^∗^	0.84^∗^	0.80^∗^	0.85^∗^	0.64^∗^	0.81^∗^	0.25^∗^	1						
FLL	0.66^∗^	0.68^∗^	0.62^∗^	0.69^∗^	0.76^∗^	0.74^∗^	0.73^∗^	0.44^∗^	0.82^∗^	0.19	0.70^∗^	1					
HL	0.53^∗^	0.52^∗^	0.45^∗^	0.53^∗^	0.63^∗^	0.58^∗^	0.56^∗^	0.41^∗^	0.65^∗^	0.12	0.57^∗^	0.66^∗^	1				
TL	0.65^∗^	0.64^∗^	0.63^∗^	0.64^∗^	0.76^∗^	0.72^∗^	0.70^∗^	0.44^∗^	0.77^∗^	0.27^∗^	0.74^∗^	0.76^∗^	0.56^∗^	1			
CaC	0.60^∗^	0.63^∗^	0.63^∗^	0.67^∗^	0.83^∗^	0.81^∗^	0.82^∗^	0.60^∗^	0.80^∗^	0.32^∗^	0.80^∗^	0.75^∗^	0.64^∗^	0.75^∗^	1		
HaL	0.02	- 0.07	- 0.01	0.02	- 0.17	- 0.13	- 0.13	0.02	- 0.04	0.01	- 0.10	- 0.08	- 0.05	- 0.20	- 0.17	1	
BW	0.65^∗^	0.61^∗^	0.66^∗^	0.64^∗^	0.84^∗^	0.82^∗^	0.99^∗^	0.67^∗^	0.81^∗^	0.20	0.84^∗^	0.73^∗^	0.55^∗^	0.69^∗^	0.82^∗^	- 0.14	1

^∗^ The correlation is significant at the level of 0.01. Head length is denoted by HL, head height is denoted by HH, ear length is denoted by EL, ear width is denoted by EW, muzzle length is denoted by ML, muzzle circumference is denoted by MC, body length is denoted by BL, scapuloischial length is denoted by SIL, chest circumference is denoted by CC, hip width is denoted by HW, wither height is denoted by WH, ischium width is denoted by IW, chest depth is denoted by CD, front leg length is denoted by FLL, hind leg length is denoted by HnL, tail length is denoted by TL, cannon circumference is denoted by CaC, ham circumference is denoted by HC, hair length is denoted by HaL, and body weight is denoted by BW.

#### 3.2.3 Phenotype variability of local pig ecotypes

**Figure 5:**
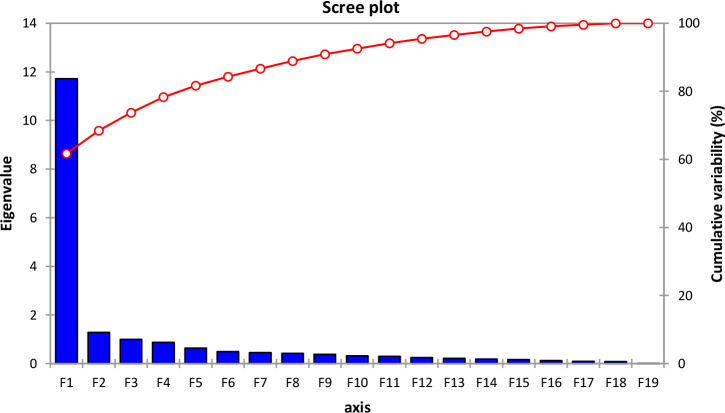
Distribution of eigenvalue cumulative variability as a function of factors.

From Table 8 and Fig. 5, 3 components out of 19 explain most of the variability at 61.69 %, 6.75 % and 5.25 % for a cumulative variability of 73.70 %. These percentages correspond to eigenvalues of 11.72, 1.28 and 0.99. This information implies that if data are represented on two axes then 73.70 % of the total variability will be preserved. For the F1 factor, four variables mostly contributed above 7 % each to the observed variability, including, by order of importance, body length (7.37), wither height (7.28), chest circumference (7.15) and cannon circumference (7.11) (Table S1 in the Supplement).

**Table 8:** Eigenvalues.

	F1	F2	F3
Eigenvalue	11.72	1.28	1.00
Variability (%)	61.69	6.75	5.26
Cumulative %	61.69	68.45	73.70

#### 3.2.4 Biometric structure of local pig population

**Figure 6:**
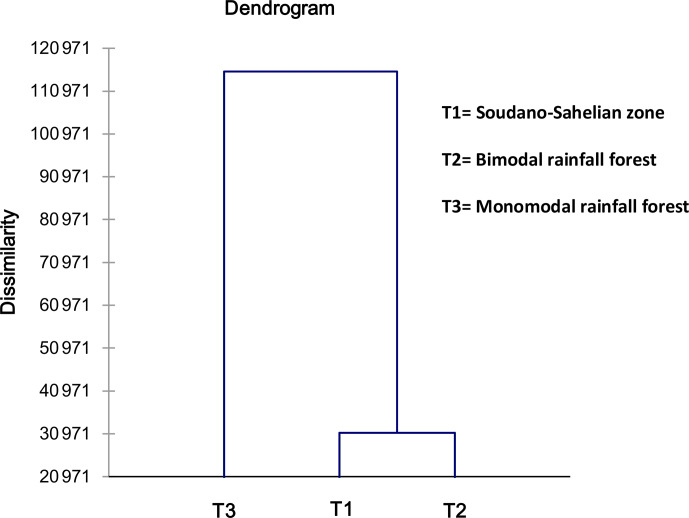
Dendrogram of local pig subpopulations in three agroecological zones.

Figure 6 is the dendrogram of the biometric structure of the local pigs from the three agroecological zones based on dissimilarity in terms of Euclidean distance using Ward's method. The dendrogram presented three subpopulations (T1, T2, T3).

**Figure 7:**
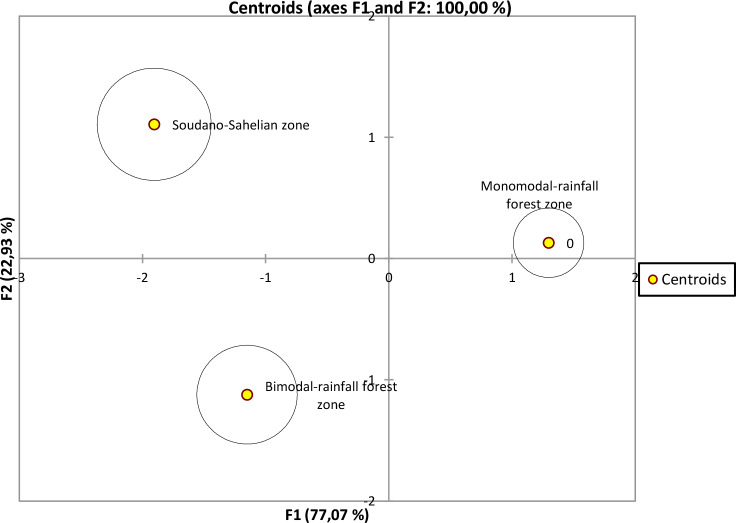
Discriminate analysis representation of F1 and F2 axes of T1, T2 and T3 based on their barycentre distance.

It clearly appears to be the fact that T1 and T2 are not similar but are closer to each other compared to T3 (Table 9 and Fig. 6). The discriminate analysis confirmed a differentiation of the three subpopulations (Fig. 7) displayed according to the F1 and F2 axes, with significant admixture evidenced among T1, T2 and T3 (Fig. S1 in the Supplement).

**Table 9:** Distance between the barycentres of agroecological zones.

	T1	T2	T3
T1 (SSZ)	0		
T2 (BRZ)	35.61	0	
T3 (MRZ)	45.91	80.96	0

BRZ denotes the bimodal-rainfall forest zone, MRZ denotes the monomodal-rainfall forest zone, and SSZ denotes the Sudano–Sahelian zone.

**Table 10:** Variance decomposition for optimal classification.

	Absolute	Percent
Within population	429.76	30.79 %
Between populations	966.23	69.21 %
Total	1396.00	100.00 %

A lower variance (30.79 %) is observed within a population, suggesting an important level of breed homogeneity or selection. The higher between-population variance component (69.21 %) indicates a higher polymorphism rate among the three pig subpopulations of Cameroon.

## 4 Discussion

This study revealed a great variety of coat colours as an expression combination of primary and secondary coat colour genes, as well as of modifiers and dilutors. The great variety of coat colours has been also evidenced by Djimenou et al. (2018), Siewe et al. (2021), Ghomsi et al. (2022) and Zhong et al. (2022), respectively, in Benin, Cameroon, Cameroon and China. However, this study is the first of it kind with regard to the phenotyping and coding of the phenotypic-diversity information as previous literature on this topic has not been found. Earlier reports from our predecessors revealed that the native pig from Cameroon is primarily black in terms of coat colour (Motsa'a et al., 2017). Therefore, the diversity in terms of coat colours observed in this study suggests that local pigs in Cameroon may have benefited from crosses with exotic breeds, including Black Hampshire, Berkshire, Yorkshire, Large White, Landrace, Piétrain and Duroc, as confirmed by their coat pattern characteristics in the described samples. This statement is in agreement with the report of AU-BIRA (2016), in which it was stated that cross-breeding of local and European breeds was the common genetic improvement practice across Africa, including in Cameroon. As evidence, crossbreeding of Black Hampshire, Large White and local pigs from the Bamboutos resulted in the black and white Bamileke pig, with white patterns from the Large White breed. Furthermore, the Sudano–Sahelian pig was obtained by crossing two exotic breeds, namely Yorkshire and Berkshire, with the indigenous pig of the far north (Mopate et al., 2006).

Coat colour patterns influenced by major genes can influence the production performance of pig breeds. In this regard, evidence suggests that coat colour genes have an impact on production and reproduction traits, with white pigs showing good reproductive performance (Li et al., 2022). On the other hand, the dwarf pig of Ghana, characterised by its black coat colour, is endangered, with exceptional hardiness and disease-resistant merit (Amponsah et al. 2017). Despite these reports on the correlation between white coats and reproductive traits and white coats and hardiness, more investigations are needed to confirm these conclusions as the white coat colour could also have been a choice in selective breeding for reasons of aesthetics and market trends guided by consumer preferences.

The high proportion of white coats (34.21 %) could be justified for two reasons. Primarily, it could be the result of the introgression of Large White or Landrace breeds into local pig populations for their reproduction- and growth-based genetic merits in relation to the rest of the studied population. Secondly, it may suggest that Cameroonian consumers prefer white pigs. This contradicts the assertions by Choy et al. (2002), who state that, in Korea, as in many other Asian countries, consumers prefer coloured pigs to monochrome ones.

A total of 14 morphotypes have been described, and a CL-5N coding system has been developed for information sharing with regard to phenotype diversity in pigs. This study cannot pretend to have exhausted the existing morphotype in Cameroon due to the limited sample size per zone and the limited study coverage (three zones out of five). The strong involvement of the MC1R alleles (75 %) in the colour determinism of Cameroon pigs converges with the assertions of many authors that MC1R is one of primary major genes regulating the synthesis of eumelanin (black and brown) and phaeomelanin (red and yellow) in mammalian melanocytes (Fontanesi and Russo, 2013; Legault and Chardon, 2000; Kijas et al., 1998). The E^p^ (0.75) allele of the MC1R gene has a higher frequency than that obtained by Carrion et al. (2003) in Iberian pigs (0.41), while the e (0.00) allele of the MC1R has a lower frequency compared to that obtained by the above-mentioned authors (0.31). This is explained by the fact that those alleles are characteristic of Iberian pigs, notably white, spotted and red pigs, and, therefore, constitute the origin of the introgression of both alleles into Cameroonian pig populations. The predominance of white and red coat colours in Iberian pigs is confirmed by Zhong et al. (2022).

Coat-colour-related genotypes are mendelian traits with a visible effect on the phenotype. This made it easy to identify the existing genotypes of the local pigs of Cameroon. Different combinations of the same colour patterns were observed. For example, different combinations of black, white and brown colours were observed in J, K, L and M (Table 2 and Fig. 4). Hur et al. (2013) observed white-spotted black coats and black-spotted white coats as two combinations of black and white resulting from the crossing of pure-black Korean native pigs and white Landrace pigs. This evidence shows that both combinations are rooted in the same coat-colour-based genotype. The difference in terms of their distribution across the body is probably the result of the difference in coat colour gene interactions. Fontanesi (2022) reported that pig breeds can be determined by the combination of hair colour, skin colour, and the bodily position of the pigmented regions of hair and skin. Therefore, a detailed description of genes and their mutations affecting coat colour and pigmentation in pigs will be a step toward selective breeding in Cameroon.

An analysis of biometric data was carried out and compared with the analyses of other authors. It was found that the head length results of this study were greater than those of Siewe et al. (2021) and Adeola et al. (2013), whereas the ear length, body length and muzzle length were lower than those recorded by Siewe et al. (2021) and greater than those of Adeola et al. (2013). The structuring of the local pigs of Cameroon into subgroups according to their agroecological zones as done in this study matches with the studies of Motsa'a et al. (2021) and Djimenou et al. (2018) on local pigs from Cameroon and Benin, respectively. The studied pig population can therefore be considered to be made up of distinct ecotypes based on their biometric structure as influenced by environmental factors.

The level of introgression of the E^p^ allele responsible for the white coat colour and black spots in pigs bred in Cameroon, with a frequency of 75 % in the studied subpopulations, is sufficient evidence that the local breed is under threat of genetic absorption since this allele is typical of commercial pigs such as the Large White, Landrace, Piétrain and Berkshire breeds, which are exotic breeds. This observation concurs with that in the report of AU-BIRA (2016) which states that local African breeds are constantly threatened by genetic erosion phenomena.

## 5 Conclusion

The aim of this study was to describe the local pigs of Cameroon and to develop a standard method for reporting phenotypic-diversity information. The study revealed that pigs bred in Cameroon have a multitude of coat colours, reflecting crossbreeding with commercial breeds. In addition, the MC1R gene is the primary coat colour determiner and plays a major role in the coat colouration of pigs bred in Cameroon. The agroecological zone (environmental factor) influences the biometric structure of the local pigs of Cameroon, separated into subgroups or ecotypes. The CL-5N coding system developed in this study is innovative and will standardise phenotypic information sharing within the scientific community and between stakeholders for a harmonised record system of genetic information, breed reporting and conservation information.

## Supplement

10.5194/aab-68-239-2025-supplement The supplement related to this article is available online at https://doi.org/10.5194/aab-68-239-2025-supplement.

## Data Availability

The data presented in this study are available upon request from the corresponding author.

## Sample availability

In this study, samples were not collected from animals in the field. The phenotype descriptions were obtained through the cooperation and consent of the farmers, ensuring ethical access to the animals involved.
