# Supplementary material for: Coat-colour-related genotypes, phenotyping and biometric assessment of three ecotypes of pigs in Cameroon
Source: Arch Anim Breed. 2025 Mar 28;68(1):239–51. doi: 10.5194/aab-68-239-2025 (PMC13271531; doi:10.5194/aab-68-239-2025)
Supplement: The supplement related to this article is available online at https://doi.org/10.5194/aab-68-239-2025-supplement. [file aab-68-239-2025-supplement.zip › Table S1.pdf]

Table S1: Contribution of the variables (%)

	F1	F2	F3	F4	F5	F6	F7	F8	F9	F10	F11	F12	F13	F14	F15	F16	F17	F18	F19
Head length	5.0348	6.4482	0.7610	2.8108	10.8412	1.6472	11.4452	6.7974	0.4812	3.2996	27.3663	1.0865	11.0810	6.8377	0.0243	0.6191	2.9985	0.2066	0.2137
Ear Length	4.8126	0.2184	0.0402	9.5813	0.0003	38.4024	2.1233	8.0307	16.8648	9.7957	0.7981	0.0910	0.8035	7.5487	0.0005	0.6375	0.0539	0.0492	0.1479
Muzzle Length	4.9223	3.0216	1.5743	2.7679	20.3532	0.2115	0.8827	13.4269	4.8953	8.2421	27.5266	0.3896	9.7544	1.1896	0.1193	0.0605	0.4989	0.0165	0.1468
Muzzle circumference	4.8714	3.9749	0.6631	1.5901	1.6231	27.8858	12.1079	17.3314	0.7694	15.1342	1.4742	0.4130	11.0071	0.0000	0.0272	0.0959	0.9575	0.0531	0.0206
Body Length	**7.3761**	0.1365	0.9840	0.0012	0.7696	0.0529	0.0383	0.0466	0.8352	4.8099	0.7772	9.3759	5.7100	5.0638	0.0321	0.1610	5.7450	58.0800	0.0048
Scapulo-Ischial Length	6.9960	0.0158	0.2281	0.0115	1.1058	1.5730	0.0968	0.7078	4.0644	6.4074	0.1634	14.2734	10.4802	14.6041	3.0603	5.8798	7.2282	23.0698	0.0342
Chest circumference	**7.1487**	0.0426	1.1921	5.3911	1.9401	0.0049	0.8239	0.2971	0.8212	3.0533	1.3387	23.1474	0.5030	1.5673	1.8266	0.0163	0.7330	0.1084	50.0444
Hip width	3.7435	0.7861	0.1429	39.5645	0.0120	2.8763	14.0006	11.2287	2.1849	14.5030	2.4454	1.3829	2.2807	4.6330	0.0437	0.0003	0.1702	0.0011	0.0003
Withers Height	**7.2787**	0.8962	0.0070	0.6349	0.0540	0.4708	0.0255	0.0115	0.7203	1.8553	1.1953	0.1889	0.0372	1.8295	16.1304	63.9225	2.6476	2.0650	0.0294
Ischium Width	0.8425	0.3386	84.7495	0.2195	0.3388	0.1306	2.9060	0.1762	5.3642	1.7660	0.0740	1.5156	0.0147	0.1604	0.0051	0.9533	0.0251	0.4026	0.0173
Chest Depth	6.8349	0.0255	0.1630	2.7688	0.6412	2.0808	0.1821	1.4512	0.3437	7.2029	1.4473	3.9416	2.8289	11.2117	39.1445	9.8964	6.1027	3.6866	0.0460
Front Leg length	6.3133	0.1483	0.6780	7.0067	3.5838	0.0441	2.6810	0.0528	0.2445	0.2204	7.0419	11.4996	9.8299	33.9752	1.3520	7.3379	7.8303	0.1323	0.0280
Hindleg length	4.1722	0.3527	3.2094	4.7401	35.5657	0.7838	36.4225	0.1314	0.0004	0.4010	10.1580	0.7876	0.2019	0.9430	0.0980	0.6574	1.3727	0.0000	0.0023
Tail Length	5.9701	0.3388	0.3365	7.9057	0.9408	0.0482	0.0262	7.5066	15.2295	9.3556	10.9624	2.4508	11.5754	3.9550	7.4791	0.1245	14.2501	1.5449	0.0001
Cannon circumference	**7.1116**	1.0864	0.3900	0.9923	3.3915	0.3429	0.9519	0.7838	0.0990	0.2122	0.0725	3.7935	2.0084	2.7768	24.6228	8.5826	36.3233	6.4560	0.0024
Ham circumference	5.3000	2.5349	0.0335	5.6288	7.7938	3.6487	5.8966	23.5166	9.6051	5.0720	4.1897	2.6126	5.7315	2.3442	0.1196	0.5828	11.9526	3.4368	0.0003
Longueur des poils	0.1737	62.2388	0.0928	2.4446	6.6587	7.4898	6.4698	3.8243	5.6909	2.7915	0.0411	0.1661	0.2798	0.5440	0.2194	0.2547	0.0599	0.5530	0.0070
Hair Length	3.9983	17.2444	3.6375	0.0064	2.6526	12.2984	1.2660	4.6183	31.7118	2.9706	0.0383	0.4585	15.4066	0.4522	2.9860	0.1707	0.0022	0.0046	0.0769
Body weigth	**7.0994**	0.1512	1.1173	5.9339	1.7340	0.0080	1.6538	0.0608	0.0743	2.9072	2.8896	22.4255	0.4659	0.3638	2.7092	0.0469	1.0484	0.1334	49.1775
