# Supplementary material for: Coat-colour-related genotypes, phenotyping and biometric assessment of three ecotypes of pigs in Cameroon
Source: Arch Anim Breed. 2025 Mar 28;68(1):239–51. doi: 10.5194/aab-68-239-2025 (PMC13271531; doi:10.5194/aab-68-239-2025)
Supplement: The supplement related to this article is available online at https://doi.org/10.5194/aab-68-239-2025-supplement. [file aab-68-239-2025-supplement.zip › Figure S1.pdf]

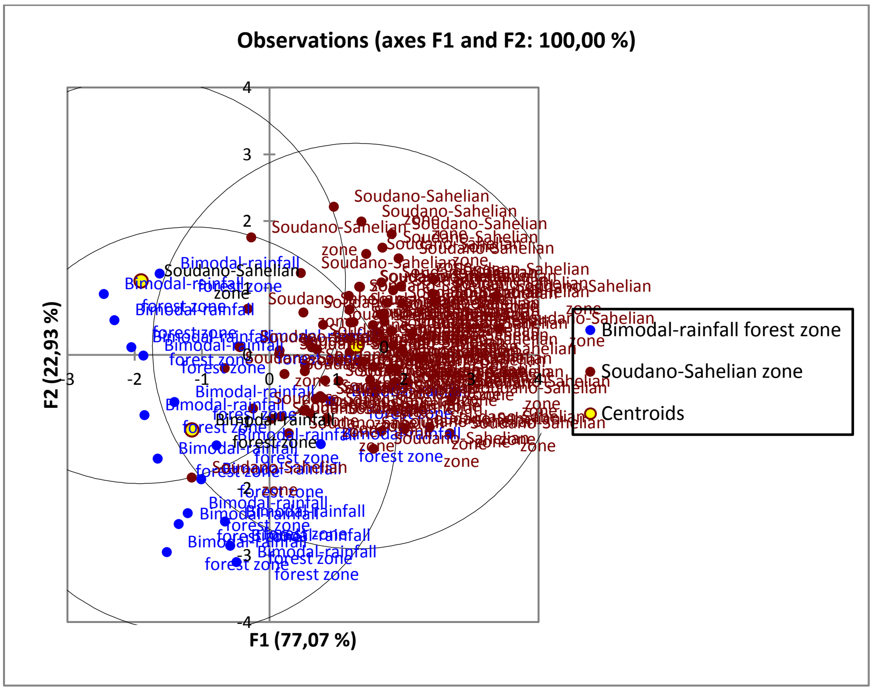

Figure S1: Discriminate analysis showing phenotypic admixture among the pigs from the three agroecological zones on F1 and F2 axes
