# Supplementary material for: Coat-colour-related genotypes, phenotyping and biometric assessment of three ecotypes of pigs in Cameroon
Source: Arch Anim Breed. 2025 Mar 28;68(1):239–51. doi: 10.5194/aab-68-239-2025 (PMC13271531; doi:10.5194/aab-68-239-2025)
Supplement: The supplement related to this article is available online at https://doi.org/10.5194/aab-68-239-2025-supplement. [file aab-68-239-2025-supplement.zip › aab-68-239-2025-supplement-title-page.pdf]

Supplement of Arch. Anim. Breed., 68, 239–251, 2025
https://doi.org/10.5194/aab-68-239-2025-supplement
© Author(s) 2025. CC BY 4.0 License.

Open Access
Archives Animal Breeding

Supplement of

Coat-colour-related genotypes, phenotyping and biometric assessment of three ecotypes of pigs in Cameroon

Aveniol Idelo Nono Ekane et al.

Correspondence to: Blaise Arnaud Hako Touko (hakoarnaud@gmail.com)

- aab-68-239-2025-supplement-title-page.pdf
- Figure S1.pdf
- Table S1.pdf

The copyright of individual parts of the supplement might differ from the article licence.
